# PdMo nanoflowers for endogenous/exogenous-stimulated nanocatalytic therapy

**DOI:** 10.3389/fphar.2023.1324764

**Published:** 2023-12-07

**Authors:** Xinqiang Liang, Yanping Tang, Mekhrdod S. Kurboniyon, Danni Luo, Guiwan Tu, Pengle Xia, Shufang Ning, Litu Zhang, Chen Wang

**Affiliations:** ^1^ Department of Research and Guangxi Cancer Molecular Medicine Engineering Research Center and Guangxi Key Laboratory of Basic and Translational Research for Colorectal Cancer, Guangxi Medical University Cancer Hospital, Nanning, China; ^2^ National Academy of Sciences of Tajikistan, Dushanbe, Tajikistan

**Keywords:** PdMo nanoflowers, ROS, peroxidase-like activity, tumor therapy, GSH

## Abstract

The clinical application of reactive oxygen species (ROS)-mediated tumor treatment has been critically limited by inefficient ROS generation. Herein, we rationally synthesized and constructed the three-dimensional PdMo nanoflowers through a one-pot solvothermal reduction method for elaborately regulated peroxidase-like enzymatic activity and glutathione peroxidase-like enzymatic activity, to promote oxidation ROS evolvement and antioxidation glutathione depletion for achieving intensive ROS-mediated tumor therapy. The three-dimensional superstructure composed of two-dimensional nanosheet subunits can solve the issues by avoiding the appearance of tightly stacked crystalline nanostructures. Significantly, Mo is chosen as a second metal to alloy with Pd because of its more chemical valence and negative ionization energy than Pd for improved electron transfer efficiencies and enhanced enzyme-like activities. In addition, the photothermal effect generated by PdMo nanoflowers could also enhance its enzymatic activities. Thus, this work provides a promising paradigm for achieving highly ROS-mediated tumor therapeutic efficacy by regulating the multi-enzymatic activities of Pd-based nanoalloys.

## 1 Introduction

Cancer is a malignant tumor that grows uncontrollably due to mutations in the genetic material of cells in the body (Yang et al., 2019; Llop and Lammers, 2021; Diao and Liu, 2023). There are more than 100 different types of cancer and it can occur in almost any part of the body. Due to the characteristics of infinite proliferation, abnormal differentiation, invasive growth, and immune escape, the treatment of cancer is extremely difficult, which brings a serious burden to society and patients. The existing clinical tumor treatment methods mainly include surgical resection, chemotherapy, radiotherapy, and immunotherapy. Still, these methods have their limitations and cannot meet the needs of patients (Tan et al., 2022; Zhang et al., 2022; Yang F. et al., 2023; Zhang L. et al., 2023; Liu et al., 2023; Overchuk et al., 2023). Surgical failure to resect tumor tissue completely is likely to lead to recurrence and metastasis, systemic toxicity and drug resistance caused by chemotherapy drugs, toxic side effects caused by high doses of radiation on normal tissues, and individual differences leading to low efficiency of the immune response. Therefore, developing multifunctional therapeutic systems to circumvent their side effects and improve anti-cancer efficacy is of great significance for the application of tumor biology.

Nanomaterials have different properties, including optical, magnetic, thermal, and catalytic properties, which show great potential in cancer treatment (Zhen et al.; Zhang et al.; Yang D. et al., 2023; Chen et al., 2023; Lin et al., 2022; Chandra and Hu, 2022; Zhou et al., 2021; Xu and Pu, 2021; Xujiang et al., 2021; Wang et al., 2021). First, nanomaterials can passively target tumors through the enhanced permeability and retention effect of solid tumors (Liu et al., 2022; Li et al., 2023; Song et al., 2023; Tang et al., 2023). Secondly, nanomaterials are easy to modify by therapeutic agents such as targeting molecules, reactive molecules and drugs, nucleic acids, peptides, and proteins to further improve targeting ability, endocytosis, and anticancer effects (Wang et al., 2020; Teng et al., 2021; Wu et al., 2022; Wang W. et al., 2023; Wang Y. H. et al., 2023). Third, a stimulus-responsive drug delivery system based on nanomaterials is constructed, which can realize the fixed-point release of drugs according to endogenous/exogenous stimuli (including hydrogen peroxide, acidity, light, magnetic field, ultrasound, *etc.*), avoid the side effects of drugs, and improve the antitumor efficacy (Kon et al., 2021; Chen et al., 2022; Kang et al., 2022; Zhang H. et al., 2023; Ji et al., 2023; Yuan et al., 2023).

Reactive oxygen species (ROS) are a class of small molecule products after oxygen with electrons, with high activity, including oxygen radicals and oxygen-containing compounds (Kankala et al., 2017; Xie et al., 2020; Wu et al., 2021; Dong et al., 2022; Li et al., 2022; Guo et al., 2023; Huang et al., 2023). ROS plays an important role in maintaining normal cell function at the physiological level. It can not only act as a signaling pathway-related regulator to regulate immune response and autophagy process but also have significant effects on cell growth, chemical reaction regulation, and gene activation (Ju et al., 2016; Liming et al., 2021; Niu et al., 2021; Peng et al., 2022). However, when ROS exceeds normal physiological levels, oncogenic transformation can occur by inducing DNA damage in normal cells and genomic instability. At the same time, studies have found that ROS not only has a multifaceted role in tumorigenesis but also changes in its level in cancer-initiating stem cells are also one of the important causes of tumor recurrence. Superoxide anion, hydroxyl radical, hydrogen peroxide (H_2_O_2_), lipid peroxide, *etc.*, are ROS. In addition, ROS is continuously generated by xanthine oxidase, lipoxygenase, and iron-catalyzed Fenton reactions. Nowadays, the combination of nanotechnology and “ROS science” for tumor therapy has extraordinary experimental results, such as photodynamic therapy, chemodynamic therapy, and sonodynamic therapy.3, (Feng et al., 2017; Zhu et al., 2020; Shueng et al., 2021; Pan et al., 2022; Truong Hoang et al., 2022; Wang et al., 2022; Jo et al., 2023).

Herein, we rationally synthesized and constructed the three-dimensional PdMo nanoflowers through a one-pot solvothermal reduction method for elaborately regulated peroxidase-like enzymatic activity and glutathione peroxidase-like enzymatic activity, to promote oxidation ROS evolvement and antioxidation glutathione depletion for achieving intensive ROS-mediated tumor therapy. In this work, the three-dimensional superstructure composed of two-dimensional nanosheet subunits can solve the issues by avoiding the formation of tightly packed crystalline nanostructures. Significantly, Mo is selected as a second metal to alloy with Pd because of its more chemical valence and negative ionization energy than Pd for improved electron transfer efficiencies and enhanced enzyme-like activities. In addition, the photothermal effect generated by PdMo nanoflowers could also enhance its enzymatic activities. In a word, this article presents a significant application of Pd-based nanoalloys with rationally designed structure and POD-like enzymatic activities for achieving intensive ROS-mediated tumor therapeutic efficacy.

## 2 Experimental section

### 2.1 Chemicals and materials

Sodium tetrachloropalladate (Na_2_PdCl_4_, 99%), molybdenyl acetylacetonate (C_10_H_14_MoO_6_), tungsten hexacarbonyl (W(CO)_6_, 99%), acetic acid (C_2_H_4_O_2_), N,N-dimethylformamide (DMF), mPEG-NH_2_, 3,3′,5,5′-tetramethyl-benzidine (TMB), glutathione (GSH), dimethyl sulfoxide (DMSO), Rhodamine B (RhB), 5,5-dithiobis-(2-nitrobenzoic acid) (DTNB), polyethylene glycol (PEG) and ethanol were obtained from Sigma-Aldrich (Shanghai, China). 2′,7′-dichlorofluorescein diacetate (DCFH-DA), propidium iodide (PI), Calcein acetoxymethyl ester (Calcein AM), 4′,6-diamidino-2-phenylindole (DAPI), methyl thiazolyl-diphenyl-tetrazolium bromide (MTT), JC-1 staining kit, ActinGreen, and Hematoxylin and Eosin Staining Kit (H&E) were obtained from Beyotime Inst. Biotech. (Haimen, China).

### 2.2 Synthesis of PdMo nanoflowers

In a typical synthesis, 0.034 mmol Na_2_PdCl_4_, 0.034 mmol MoO_2_(acac)_2_, and 0.142 mmol W(CO)_6_ were dissolved in 2 mL acetic acid and 8 mL DMF. The mixture solution was stirred and sonicated at least for 10 min. Then, the obtained suspension solution was continually heated at 140°C for 2 h. The obtained black colloidal products were centrifuged (12,000 rpm, 15 min) and washed with ethanol three times, and finally dispersed in water obtaining PdMo nanoflowers. For further bio-application, the PdMo nanoflowers were conducted biocompatible modification. The mPEG-NH_2_ and PdMo nanoflowers were dissolved in a mixed solvent of ethanol and cyclohexane for 9 h under sonication. After washing with ethanol and deionized water, the PEG-modified PdMo nanoflowers were obtained by centrifugation.

### 2.3 Characterization method

The X-ray diffraction (XRD) patterns were conducted under Cu-Ka radiation (λ = 0.154 nm) at 40 kV and 40 mA using a Rigaku D/max-TTR-III diffractometer. The structure of as-synthesized materials was recorded using transmission electron microscopy (TEM) and high-resolution TEM with an FEI Tecnai G2 S-Twin transmission electron microscope (acceleration voltage 200 kV). X-ray photoelectron spectroscopy (XPS) spectra were recorded with an ESCALAB 250 instrument. A UV-1601 spectrophotometer was used to obtain the ultraviolet-visible (UV-vis) absorbance spectrum. The element quantitative analysis of the as-synthesized samples was recorded on inductively coupled plasma-optical emission spectrometry (ICP-OES) using Agilent 725 (Agilent Technologies). Leica TCS SP8, a confocal laser scanning microscope (CLSM) was operated to obtain the fluorescence image.

### 2.4 Nanozyme properties experiments

The ROS generation of PdMo nanoflowers was analyzed by using the TMB (0.2 mM) under H_2_O_2_ conditions. The absorbance properties of the supernatant were recorded by the UV-vis spectrophotometer, and the chromogenic change was also recorded. Especially, the 808 nm laser was conducted on the ROS generation properties of PdMo nanoflowers under the same conditions for evaluating the photothermal effects of PdMo nanoflowers. The PdMo nanoflowers with different concentrations were placed in several tubes with PBS (pH = 6.5), respectively. Then, TMB and H_2_O_2_ (3 mL) were added with and reacted at 37°C in the dark. The absorbance of the mixed solution was recorded at 652 nm for 10 min.

The GSH depletion properties of PdMo nanoflowers were investigated by DTNB, where GSH could react with DTNB (colorless solution) forming a yellow solution, especially showing a special absorption around 412 nm. The GSH consumption of PdMo nanoflowers was demonstrated by the weakening of 412 nm absorption. In detail, PBS solution (5.4 mL) containing PdMo nanoflowers (1.8 mg/mL, 600 μL) and GSH solution (1.8 mg/mL, 600 μL) were mixed. Then, DTNB (1 mM, 500 μL) was added into the above mixture (500 μL), while the UV-vis absorption spectrum was recorded at different times (1, 3, 5, 7, and 9 h).

### 2.5 Photothermal properties experiments

Under room temperature, the PdMo nanoflowers solution was prepared in different concentrations (12.5, 25, 50, and 100 μg/mL) and irradiated with an 808 nm NIR laser (10 min). The temperature fluctuation was measured by thermal imaging equipment at specified intervals. Especially, the pure water was conducted in the same process and used as a control group. Moreover, the photothermal stability was also investigated by irradiating the PdMo nanoflowers solution (100 μg/mL) for several cycles, and the variation of temperature was measured. According to one of the cooling curves, the photothermal conversion efficiency (*η*) of PdMo nanoflowers was determined, obtained by the following formula:

$$\eta =\frac{hA\left(T\max\;―T\text{surr}\right)―\text{Qs}}{I\left(1―10^{―\;A_{\lambda }}\;\right)}$$

where *h* is the heat transfer coefficient, *A* refers to the superficial area, *Tmax* and *Tsurr* is the maximum equilibrium temperature of the PdMo nanoflowers solution and ambient temperature during testing, respectively. *Qs* = (5.4 × 10⁻^4^)*I*, *I* refer the power density of the 808 nm laser, and *A_λ_* refer the absorption value of PdMo nanoflowers solution.

### 2.6 *In vitro* anti-tumor properties

*Cellular Uptake Assay.* The cellular uptake of PdMo nanoflowers was recorded by CLSM with the label of RhB in CT26 cells. In brief, the CT26 cells were seeded in 6-well plates and cultured at 37°C, and then RhB-labeled PdMo nanoflowers (1 mL, 200 μg/mL), which were obtained from PdMo nanoflowers incubating with RhB fluorescence, were added to the CT26 cells and incubated for 0.5, 1, 2, 3 and 5 h. The cells were stained with hoechst 33,342 to visualize the nucleus.

*Cytotoxicity Assay.* The CT26 and L929 cells (L929 fibroblast cell line) were regularly cultured in 96-well plates and cultured for standard MTT analysis. First, the L929 cells were seeded in a 96-well plate and cocultured with MTT for 12 h and 24 h, respectively, with different PdMo nanoflowers concentrations (12.5, 25, 50, 100, and 200 μg/mL). The absorbance at 490 nm of the 96-well plate was measured in a plate reader. The CT26 cells were similarly treated, and the group set as control, NIR, PdMo, and PdMo + NIR, respectively. The PdMo nanoflowers concentrations were set as 12.5, 25, 50, 100, and 200 μg/mL for PdMo nanoflowers-related groups, respectively. The laser-related groups were exposed to an 808 nm laser (0.8 W/cm^2^, 10 min).

*Living/Dead Cell Staining Assay.* The living/dead cell staining assay was measured by CLSM using calcein-AM/PI. The cells were subjected to different treatments: control, NIR, PdMo (200 μg/mL), and PdMo + NIR (200 μg/mL, 0.8 W/cm^2^, 10 min). After continued cultivation, the calcein-AM/PI co-stained with the cells. Finally, wash the CT26 cells with PBS and observe them by using CLSM.

*Mitochondrial Membrane Potential Detection.* The CT26 cells were cultured in a glass-bottom Petri dish. Then, replacing the cell medium and handling the cells with various treatments: control, NIR, PdMo, and PdMo + NIR. The NIR-related groups were exposed to an 808 nm laser with a power density of 0.8 W/cm^2^ for 10 min and co-incubation for another 4 h. The CT26 cells were further stained with JC-1 and DAPI, respectively. Finally, the cells were observed under a CLSM.

### 2.7 *In vivo* anti-tumor properties

The female BALB/c mice in 4-week-old were raised in suitable conditions with sterile water and food. The animal study protocol was approved by the Ethics Committee of Guangxi Medical University Cancer Hospital (protocol code KY 2022-129/130 and approved on 25 February 2022) for studies involving animals.

*In vivo biodistribution.* The PdMo nanoflowers (20 mg/kg) were injected into CT26 tumor-bearing BALB/c mice (n = 3). The mice were disposed at different times (1, 3, 6, 12, 24, and 48 h) and the corresponding organs (heart, liver, spleen, lung, and kidney) and tumors were collected. The content of the Mo element was detected by ICP-OES.

*In vivo antitumor efficacy.* The CT26 tumor-bearing mice model was constructed and randomly separated as four groups (Diao and Liu, 2023): control (Llop and Lammers, 2021), NIR (Yang et al., 2019), PdMo (Zhang L. et al., 2023), PdMo + NIR. The mice (tumor volume was around 100 mm^3^) were intravenously treated with PdMo nanoflowers (20 mg/kg) on days 1, 4, and 7. After 6 h, tumor sites were irradiated with 808 nm lasers (0.8 W/cm^2^) for 10 min. The body weight and tumor size of each mouse were recorded every 2 days. The tumor volume was determined as: tumor volume = (tumor length) × (tumor width)^2^/2. After 14 days, all mice were euthanized and the excised tumors were further selected for H&E staining.

### 2.8 Statistical analysis

Quantitative data were presented as mean ± standard deviation (mean ± S.D.). The software of GraphPad Prism 9.0 was adopted to assess the statistical analysis. The statistical significance attained at *p < 0.05 (significant), **p < 0.01 (moderately significant), and ***p < 0.001 (highly significant).

## 3 Result

### 3.1 Structure and morphology characterization

two-dimensional Pd nanoalloys have been widely used in biological detection due to their abundant active sites and high specific surface area. However, two-dimensional Pd nanoalloys tend to pack together resulting in low catalytic activity. Significantly, the three-dimensional superstructure composed of two-dimensional nanosheet subunits can solve the issues by avoiding the appearance of tightly stacked crystalline nanostructures. Herein, Mo is selected as a second metal element to alloy with Pd because of its more chemical valence and negative ionization energy than Pd for improved electron transfer efficiencies and enhanced POD-like enzymatic activities. Therefore, PdMo nanoflowers were rationally constructed and synthesized through a one-pot solvothermal reduction method. For further bio-application, PdMo nanoflowers were modified with PEG-NH_2_ to improve their colloidal stability in blood circulation.

**FIGURE 1:**
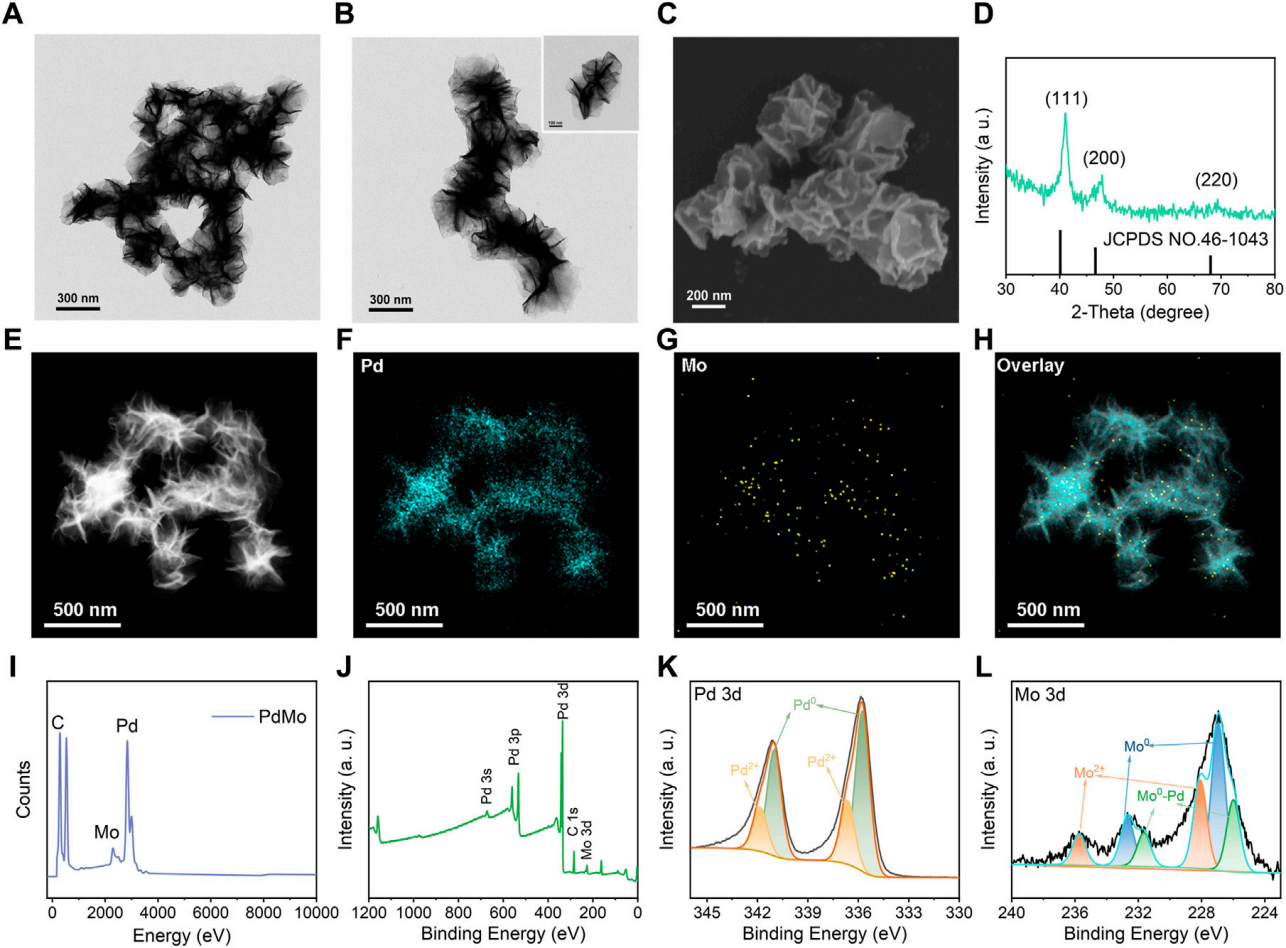
**(A, B)** The representative TEM images of PdMo nanoflowers. **(C)** The representative SEM image of PdMo nanoflowers. **(D)** XRD pattern of PdMo nanoflowers. **(E–H)** Elemental mapping of PdMo nanoflowers. **(I)** EDS spectrum of PdMo nanoflowers. **(J)** XPS spectrum of PdMo nanoflowers. **(K)** High-resolution XPS spectrum of Pd 3d of PdMo nanoflowers. **(L)** High-resolution XPS spectrum of Mo 3d of PdMo nanoflowers.

The TEM images in Figures 1A,B showed that the as-synthesized PdMo nanoflowers exhibit a typical three-dimensional flower structure, comprising of many two-dimensional cross-linked nanosheets. The average particle size was around 180 nm. The SEM images (Figure 1C) also showed the successful construction of the three-dimensional flower-like superstructure. XRD was utilized to demonstrate the crystal structure of PdMo nanoflowers (Figure 1D). It can be seen that PdMo nanoflowers showed specific diffraction peaks, corresponding to the (111) (200), and (220), of the Pd XRD pattern (JCPDS NO. 46-1043). However, due to the Mo addition, the XRD diffraction peaks of PdMo nanoflowers increasingly shift to a higher angle, indicating the lattice shrinks and PdMo nanoalloys are formed. The element mapping of PdMo nanoflowers revealed that both Pd and Mo elements were uniformly dispersed throughout the nanoflowers (Figures 1E–G). The EDS spectrum also demonstrated the presence of Mo and Pd elements (Figure 1I). To determine the surface states of Pd and Mo elements in the PdMo nanoflowers, XPS spectra of Pd 3d and Mo 3d are measured. As shown in Figure 1J, the XPS spectra of the PdMo nanoflowers clearly indicated the presence of Pd and Mo elements. Figure 1K indicated that the high resolution of Pd 3p spectrum could be divided into four peaks, where two pairs of peaks presented at 340.5 eV and 335.5 eV were attributed to Pd^0^, and the other two weak peaks at 341.8 eV and 336.9 eV were attributed to and Pd^2+^. As displayed in the Mo 3d high-resolution XPS spectrum of PdMo nanoflowers (Figure 1L), the dominant peaks at 235.8 eV and 228.0 eV corresponded to the binding energies of 3d_3/2_ and 3d_5/2_ of Mo^2+^, respectively, while the lower binding energy at 233.6 eV and 226.6 eV belonged to 3d_3/2_ and 3d_5/2_ of Mo^0^, respectively.

### 3.2 Nanozymes activities properties

**FIGURE 2:**
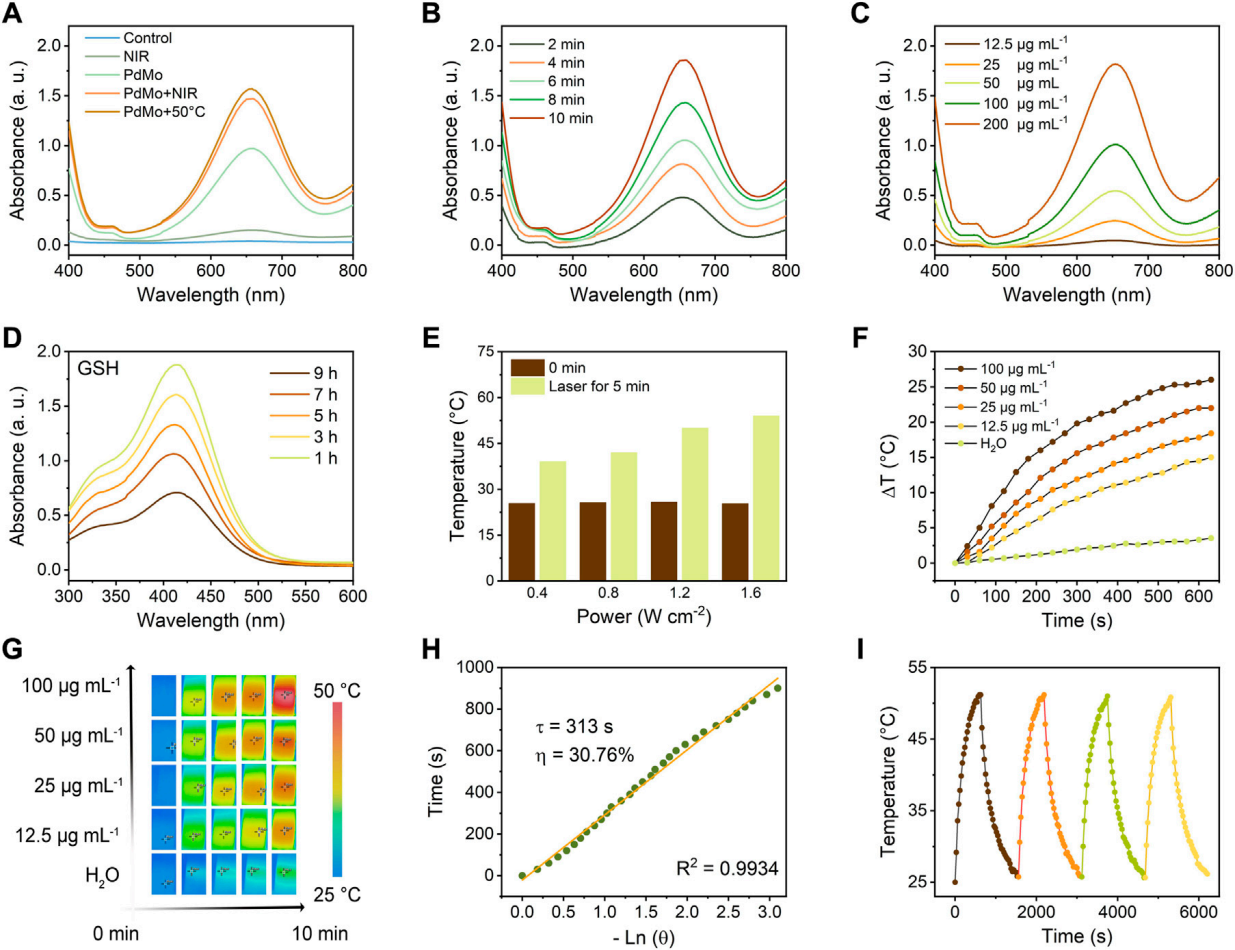
**(A)** UV-vis absorption spectra of TMB aqueous solution treated with different conditions, such as control, PdMo, NIR (0.8 W/cm^2^, 5 min), PdMo + NIR (0.8 W/cm^2^, 5 min), and PdMo+50°C. **(B)** UV-vis absorption spectrum of TMB aqueous solution (PdMo + NIR, 0.8 W/cm^2^) with different times. **(C)** UV-vis absorption spectrum of TMB aqueous solution (PdMo + NIR, 0.8 W/cm^2^, 5 min) with different concentrations. **(D)** UV-vis absorption spectrum of DTNB (PdMo + NIR) with different times. **(E)** Power density-dependent photothermal effect of PdMo nanoflowers. **(F)** Concentration-dependent photothermal curves of PdMo nanoflowers irradiated with 808 nm laser. **(G)** The corresponding IR images of (f). **(H)** The time constant (*τ*_s_ = 313 s) and photothermal conversion efficiency (30.76%) for the heat transformation. **(I)** Thermal stability of PdMo nanoflowers irradiated with 808 nm laser.

To identify the peroxidase-like enzymatic activity of PdMo nanoflowers, TMB was utilized to verify the hydroxyl radical (•OH) generation upon 808 nm laser irradiation. As shown in Figure 2A, in the presence of H_2_O_2_, PdMo nanoflowers can cause the oxidation of TMB substrates (colorless solution) to oxide TMB (blue solution) at approximately 652 nm absorbance spectra. Particularly, the TMB absorption peaks in the PdMo nanoflowers + NIR group with the presence of H_2_O_2_ after different 808 nm laser irradiation was higher than that of the PdMo nanoflowers group, proving that the POD-like catalytic activity of the PdMo nanoflowers could be dramatically enhanced by NIR irradiation. The above results demonstrate that PdMo nanoflowers exhibit POD-like enzymatic activities and could be used as promising nanozymes for ROS-mediated tumor therapy. At the 50°C condition, the absorbance spectra of oxide TMB at approximately 652 nm showed enhanced intensity. Compared with PdMo + NIR group, the photothermal effect generated by the NIR-activated PdMo nanoflower also demonstrate the above conclusion. As demonstrated in Figure 2B, the typical TMB absorption peak represented the •OH production gradually enhanced with the reaction time, verifying that the PdMo nanoflowers could exert the Fenton reaction to catalyze H_2_O_2_ into •OH. The reaction rate of TMB oxidation was also dependent on the concentration of PdMo nanoflowers solution (Figure 2C). Furthermore, we have compared the POD-like enzymatic activities of PdMo nanoflowers with the Pd nanoalloys and Fe_3_O_4_ nanozymes (200 μg/mL). As shown in Supplementary Figure S1, the as-synthesized PdMo nanoflowers exhibited the highest POD-like enzymatic activities, demonstrating the superior advantages of Mo-doping for more chemical valence and improved electron transfer, as well as the three-dimensional superstructure of tightly stacked crystalline nanostructures.

GSH, one of the primary endogenous antioxidant species, maintains the redox balance of biological systems. The overexpressed GSH in the tumor microenvironment (TME) could deplete ROS, thus resulting in the inefficiency of ROS generation. Hence, depleting GSH is a facile approach to achieving ROS accumulation for effective treatment. The GSH consumption-ability of PdMo nanoflowers was investigated based on a GSH indicator, i.e., DTNB, which could be reduced by GSH, showing the characteristic absorbance at around 412 nm absorbance. As shown in Figure 2D, in the presence of PdMo nanoflowers, the GSH content gradually decreased with time. The depletion of GSH by PdMo nanoflowers retains a relatively high level of ROS in tumor cells in the process of treatment.

Noble metal and noble metal alloy nanozymes have been devoted to the field of tumor treatment in recent years due to the adjustable POD-like enzymatic activities and excellent chemical stabilities in complex biological environments, as well as the unique optical properties derived from surface plasmon resonance effect (SPR) at the nanoscale level. Pure Pd nanoparticles have a high melting point and photothermal stability that can play a controllable local SPR effect in the infrared region, and pure Pd nanoparticles have superior near-infrared light (NIR) absorption and photothermal conversion properties. For evaluating the photothermal effects of PdMo nanoflowers, PdMo nanoflowers with different concentrations (12.5, 25, 50, and 100 μg/mL) were irradiated upon an 808 nm laser (0.8 W/cm^2^) for 10 min. The ultraviolet-visible (UV-vis) absorption spectrum of PdMo nanoflowers is in Supplementary Figure S2. The results indicated that the temperature increase depended on the irradiation time and PdMo nanoflowers concentrations (Figures 2E,F). As exhibited in Figure 2E, the temperature of PdMo nanoflowers solutions gradually elevated with the enhanced laser power density (0.4, 0.8, 1.2, and 1.6 W/cm^2^). Specifically, the final temperature of PdMo nanoflowers solutions after 10 min at different power densities elevated to 39.0, 42.2, 49.8, and 54.1°C, respectively. As exhibited in Figure 2F, the increased temperature of PdMo nanoflowers solution (100 μg/mL) was up to 26.0°C, while the temperature of the control group increased by only 3.5°C. The corresponding infrared images are shown in Figure 2G. As plotted in Figure 2H, from the cooling period of the curve, the heat transfer time determined from the cooling period was *τ*_s_ = 313 s. Especially, the photothermal conversion efficiency (*η*) of PdMo nanoflowers was finally determined as 30.76%, demonstrating that the PdMo nanoflowers possessed a good conversion of laser energy into heat energy. Then, the photothermal effect stability of PdMo nanoflowers was evaluated. As shown in Figure 2I, there was no evident change of the highest temperature during four cycles. The above results indicated the potential of PdMo nanoflowers in photothermal therapy and photothermal-enhanced ROS-mediated therapy.

### 3.3 *In vitro* anti-tumor properties

**FIGURE 3:**
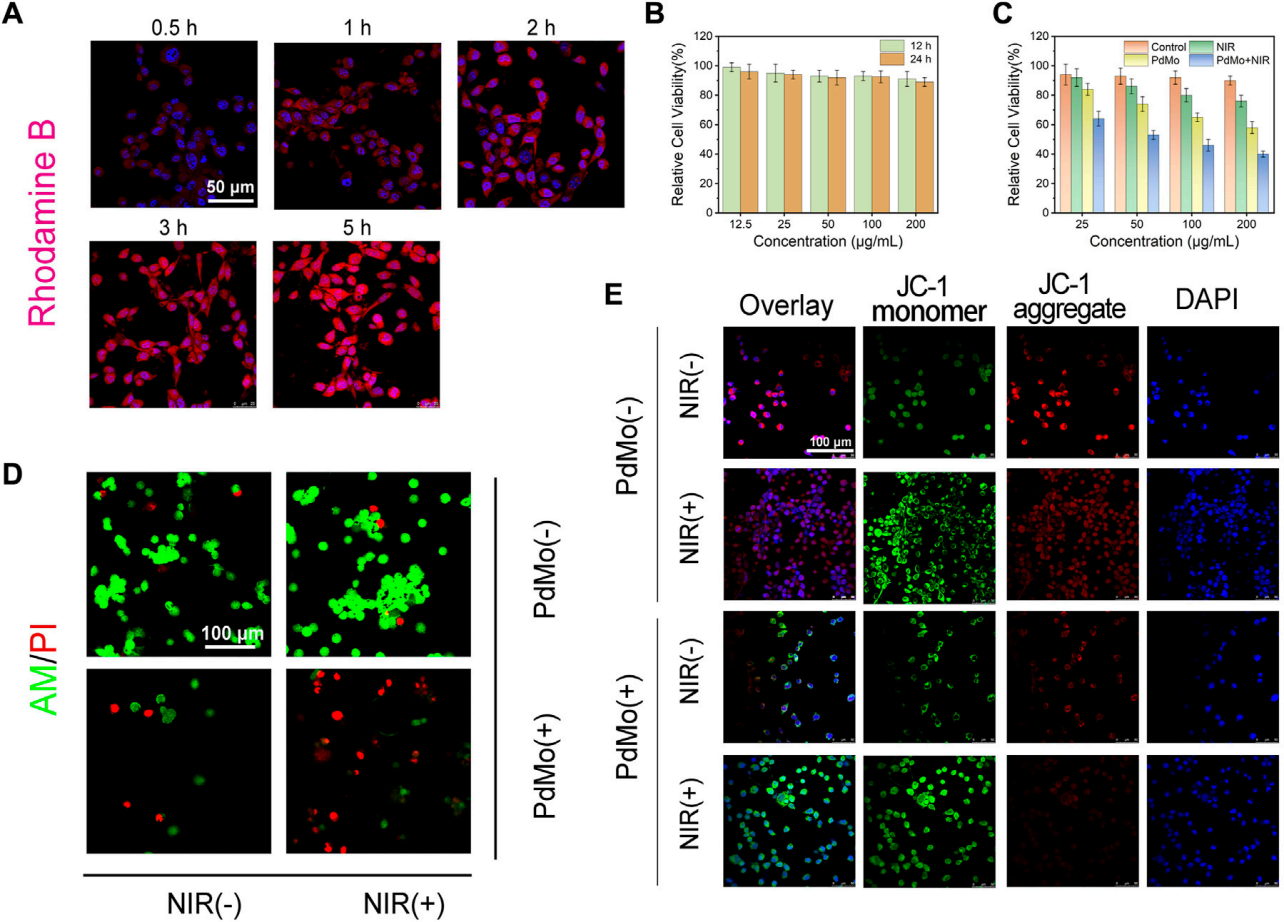
**(A)** The cellular uptake of RhB-labeled PdMo nanoflowers observed by CLSM in CT26 cells. **(B)** Relative cell viabilities of L929 cells. **(C)** Cytotoxicity assay of CT26 cells. **(D)** AM/PI staining CLSM images of CT26 cells. **(E)** JC-1 staining of CT26 cells.

The promising performance of PdMo nanoflowers makes it a potential agent for synergistic anti-tumor therapy, which was examined *in vitro* against the CT26 (mouse colon cancer cells) cell line. Before *in vivo* anti-tumor properties experiments, first, the endocytosis of PdMo nanoflowers labeled with RhB was systematically analyzed in CT26 cells. The RhB-labeled PdMo nanoflowers were incubated at different time points (0.5, 1, 2, 3, and 5 h) and observed by confocal laser scanning microscopy (CLSM). As shown in Figure 3A, the PdMo nanoflowers could enter CT26 cells easily through endocytosis, which exhibited a time-dependent manner. Only weak red fluorescence was observed, indicating only a small amount PdMo nanoflowers were endocytosed at 0.5 h. With the prolonged incubation time, the red fluorescence was enhanced, indicating that there were more PdMo nanoflowers were endocytosed. The PdMo nanoflowers entered the cell through the endolysosomal pathway. Then, we evaluated the biocompatibility and cytotoxicity of PdMo nanoflowers using standard MTT assays. Different concentrations of PdMo nanoflowers (12.5, 25, 50, 100, and 200 μg/mL) were incubated with L929 cells for 12 h and 24 h, and cell viability was calculated by the MTT method. As shown in Figure 3B, the cell activity after treatment with 0–200 μg/mL PdMo nanoflowers was 90%–100%, indicating that PdMo nanoflowers exhibited low cytotoxicity, demonstrating the excellent biocompatibility of PdMo nanoflowers for anti-tumor therapy. As shown in Figure 3C, the cell viability of PdMo nanoflowers and PdMo nanoflowers + NIR groups decreased to varying degrees, and both the increased concentration and the laser irradiation could lead to greater cytotoxicity. These results indicate that PdMo nanoflowers have higher endocytosis efficiency and good biocompatibility, suitable for anti-cancer research *in vivo*. The *in vitro* GSH depletion and ROS generation abilities of PdMo nanoflower and Pd nanoalloys were in Supplementary Figures S2, S4, respectively. The level of intracellular ROS in different treatment groups was observed by CLSM, and the level of intracellular GSH consumption in different treatment groups was measured by the GSH detection kit. As anticipated, the PdMo + NIR group exhibits the strongest green fluorescence signal, indicating a significant increase in the overall ROS content, which was facilitated by catalytic reactions and photothermal enhancement (Supplementary Figure S3). The group treated with PdMo has a higher intensity than the Pd group. The PdMo + NIR group of cells with the highest ROS levels resulted from alloying-enhanced and photothermal-enhanced POD-like enzymatic activity. The relative content of antioxidant GSH shows that the PdMo + NIR group has higher GSH consumption performance than the Pd group, and the intrinsic photothermal effect of PdMo nanoalloys improves the GSH consumption performance in PdMo + NIR group (Supplementary Figure S4).

Subsequently, we further studied the antitumor effect of the as-synthesized PdMo nanoflowers. The ability of PdMo nanoflowers to induce cell apoptosis was evaluated by the classical assay for cell apoptosis. We used calcein AM and propyl iodide (PI) as fluorescent probes to visually observe living (green) and dead cells. As can be seen from Figure 3D, no significant dead cells were detected in the control and NIR group. A minority of red dead cells were observed in the PdMo nanoflowers group. In the PdMo nanoflowers plus NIR laser group, almost all of the cells died, suggesting that upon laser irradiation, PdMo nanoflowers were active. The annexin V-FITC/PI apoptosis detection kit was utilized for binding to associated proteins within cells via flow cytometry to assess early and late apoptotic cells (Supplementary Figure S5). The CT26 cells in the PdMo + NIR group undergo apoptosis and the proportion of apoptotic in the PdMo + NIR is up to 59.6% in the Q2 + Q3 region. Moreover, the mitochondrial integrity of different groups was investigated by JC-1 staining. PdMo + NIR group presented the maximum green monomer/red aggregate fluorescence ratio, manifesting that the majority of mitochondria were damaged (Figure 3E). Together, these *in vitro* results manifest the efficient POD-like enzymatic activity as contributed by the PdMo nanoflowers-enabled ROS accumulation for anti-tumor therapy.

### 3.4 *In vivo* anti-tumor properties

**FIGURE 4:**
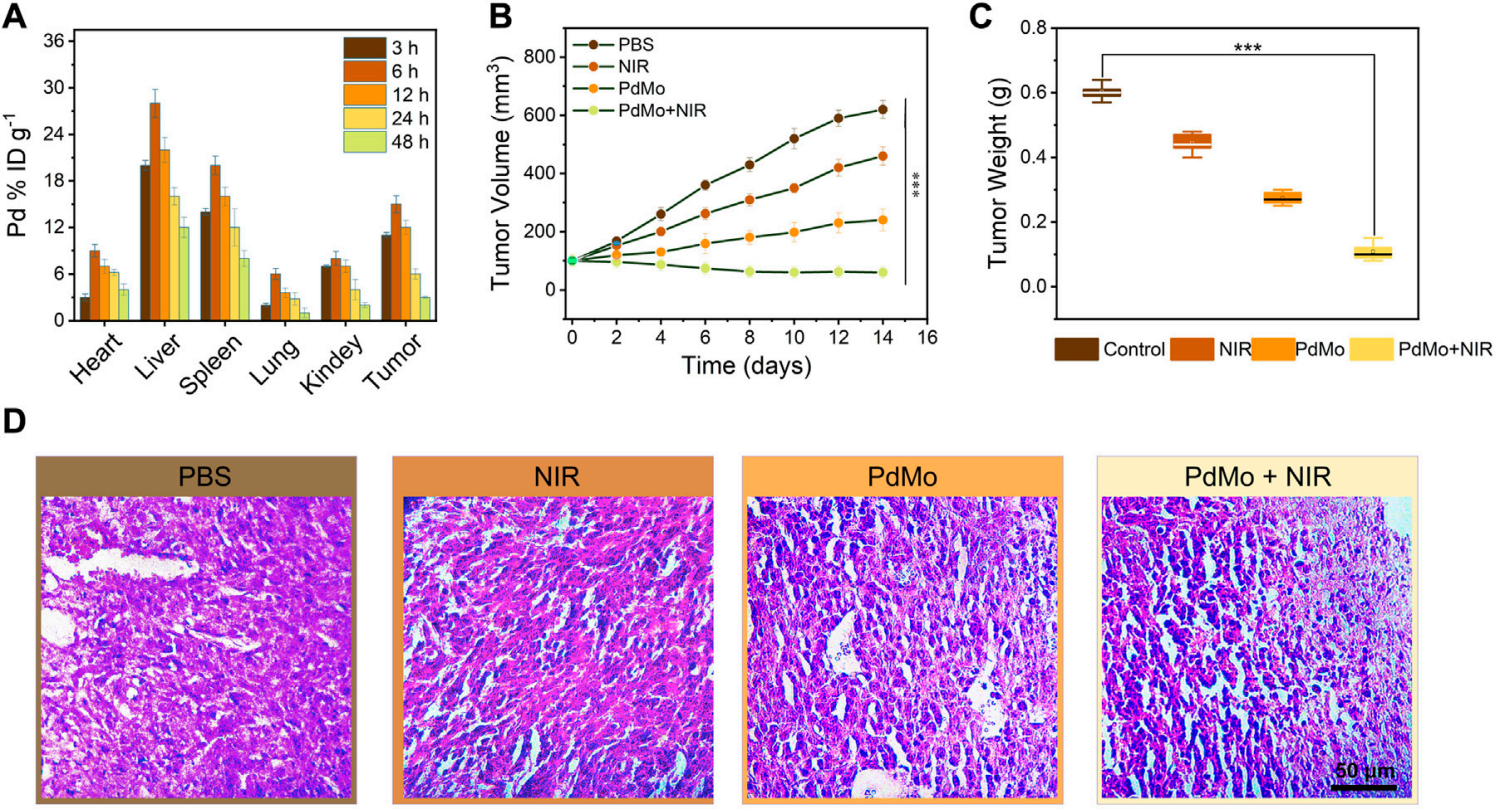
**(A)** Biodistribution of PdMo nanoflowers in tumors and main organs at various times (3, 6, 12, 24, and 48 h). **(B)** Volume and **(C)** weight changes of tumor in the mice with diverse treatments. **(D)** H&E staining of tumor slices in various groups.

Encouraged by the distinguished cooperative treatment efficacy *in vitro*, we further conducted *in vivo* experiments on CT26 tumor-bearing mice to estimate the *in vivo* anticancer therapeutic performance of the PdMo nanoflowers. First, the biodistribution of PdMo nanoflowers after i. v. injection to CT26 tumor-bearing mice was further measured to elucidate the *in vivo* behaviors. We administered intravenous infusion at different time points PdMo nanoflowers were radiated and Mo element in major organs and tumor tissues was measured by ICP-OES. As shown in Figure 4A, the liver and spleen exhibited abundant accumulation of PdMo nanoflowers due to the capture of the reticuloendothelial system. Besides, the accumulation of Mo element distributed in tumor regions achieved maximum after post-injection of PdMo nanoflowers for 6 h, and kept high concentration even at 12 h, demonstrating the efficient accumulation of PdMo nanoflowers.

To evaluate the *in vivo* anticancer therapeutic performances of the PdMo nanoflowers, twenty of CT26 tumor-bearing mice were raised in four cubicles equally (*n* = 5) and handled with diverse strategies (Diao and Liu, 2023): control (Llop and Lammers, 2021), NIR (Yang et al., 2019), PdMo (Zhang L. et al., 2023), PdMo + NIR. In a typical protocol, the tumor regions of mice in the (Llop and Lammers, 2021; Zhang L. et al., 2023) groups were irradiated by laser (0.8 W/cm^2^, 10 min) after a single i. v. administration of PdMo nanoflowers (20 mg/kg) for 6 h. During the treatment process, the body weights and tumor sizes of all mice were recorded every 2 days. As exhibited in Figures 4B,C, the control and laser groups exerted no appreciable influence on tumor growth, while the mice treated with PdMo nanoflowers exhibited an inhibitory effect on tumors. Specifically, upon 808 nm laser irradiation, the tumor growth inhibition effect in the group of PdMo + NIR could be enhanced with a suppression rate of 87%, showing similar results with *in vitro* cytotoxicity experiments. The weights of mice exhibit no significant difference among them, suggesting that no significant toxicity of the injected samples to mice during the process of treatment (Supplementary Figure S6). The *in vivo* photothermal images of PdMo nanoflowers under 808 nm irradiation were recorded in Supplementary Figure S7. The temperature of the tumor sites is raised apparently in the PdMo nanoflowers + NIR treatment group, which can achieve hyperthermia-enhanced POD-like enzymatic activity. Furthermore, the anti-tumor properties of Pd nanoalloys *in vivo* were exhibited in Supplementary Figure S8. The changes in tumor volume of mice in each group show that the PdMo group exhibits a higher tumor suppression efficiency than the Pd group, and the PdMo + NIR group shows the best tumor suppression efficiency. The weights of mice exhibit no significant difference among them, suggesting that no significant toxicity of the injected samples to mice during the process of treatment. These results collectively demonstrated the superior therapeutic effect of PdMo nanoflowers irradiated with 808 nm laser, and the impressive tumor inhibition effect mainly owing to the cooperative therapeutic efficacy including ROS generation, GSH depletion, and photothermal effect of PdMo nanoflowers. On the 15th day of treatment, all the mice were sacrificed, and the tumors of mice in various groups were dissected for staining with hematoxylin and eosin (H&E), which could be used to further validate the treatment effect of PdMo nanoflowers. As displayed in Figure 4D, the PdMo + NIR group exhibited the severest cell apoptosis and necrosis in the H&E-stained tumor sections compared with other groups in H&E staining images. Besides, biochemical blood analysis and routine examination show no significant differences in treatment groups as compared to the control group (Supplementary Figure S9).

## 4 Discussion

In summary, an endogenous/exogenous stimulus-responsive therapeutic nanoplatform of PdMo nanoflowers was constructed to afford cooperative antitumor potency of GSH-depletion/photothermal-enhanced ROS-mediated therapy. The GSH depletion ability, photothermal effects, and POD-like enzymatic activity of PdMo nanoflowers were systematically investigated and demonstrated. The promising ROS-mediated antitumor performance was also experimentally verified according to the *in vitro* and *in vivo* comparison, which is consistent with the above results. Under the acidic TME, the PdMo nanoflowers with POD-like activity could not only greatly relieve the antioxidation resistance of the tumor but also generate highly cytotoxic •OH. Moreover, the PdMo nanoflowers were capable of ideal photothermal performance, which could further improve the treatment efficiency by photothermal effect and photothermal-enhanced ROS-mediated therapy. Thus, the as-synthesized PdMo nanoflowers provide the promising potential for effective cooperative cancer therapy.

## Data Availability

The original contributions presented in the study are included in the article/Supplementary Material, further inquiries can be directed to the corresponding authors.
