# Supplementary material for: PdMo nanoflowers for endogenous/exogenous-stimulated nanocatalytic therapy
Source: Front Pharmacol. 2023 Dec 7;14:1324764. doi: 10.3389/fphar.2023.1324764 (PMC10740153; doi:10.3389/fphar.2023.1324764)
Supplement: Supplementary file 1 [file DataSheet1.docx]

Supporting Information

PdMo nanoflowers for endogenous/exogenous-stimulated nanocatalytic therapy

Xinqiang Liang^1†^, Yanping Tang^1†^, Mekhrdod S. Kurboniyon^2^, Danni Luo^1^, Guiwan Tu^1^, Pengle Xia^1^, Shufang Ning^1^, Litu Zhang^1*^, Chen Wang^1*^

^1^ Department of Research & Guangxi Cancer Molecular Medicine Engineering Research Center & Guangxi Key Laboratory of Basic and Translational Research for Colorectal Cancer, Guangxi Medical University Cancer Hospital, Nanning 530021, China;

^2^ National Academy of Sciences of Tajikistan, Dushanbe 734000, Tajikistan


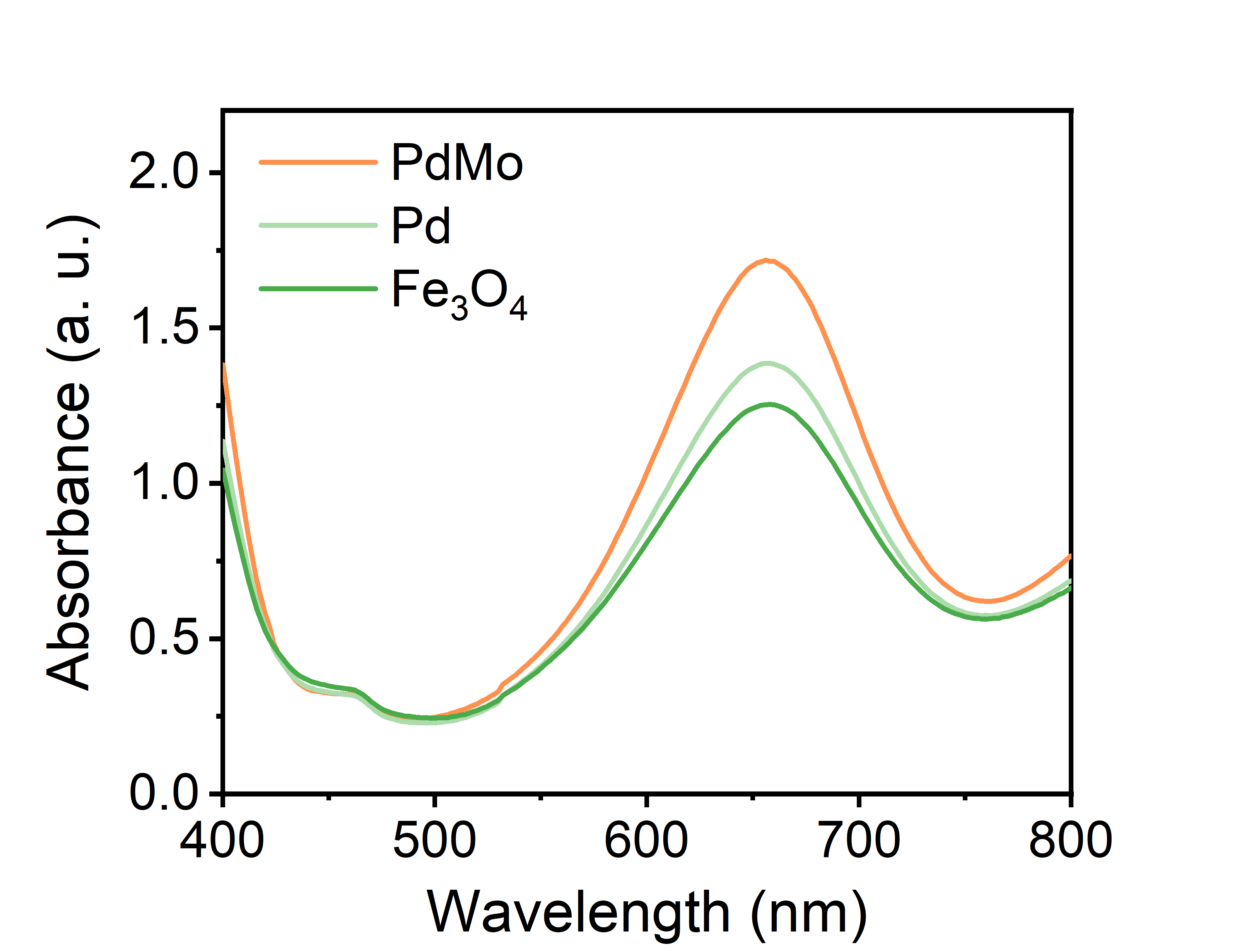


Figure S1. UV-vis absorption of PdMo nanoflowers, Pd alloys, and Fe_3_O_4_ nanoparticles (0.2 μg/mL).


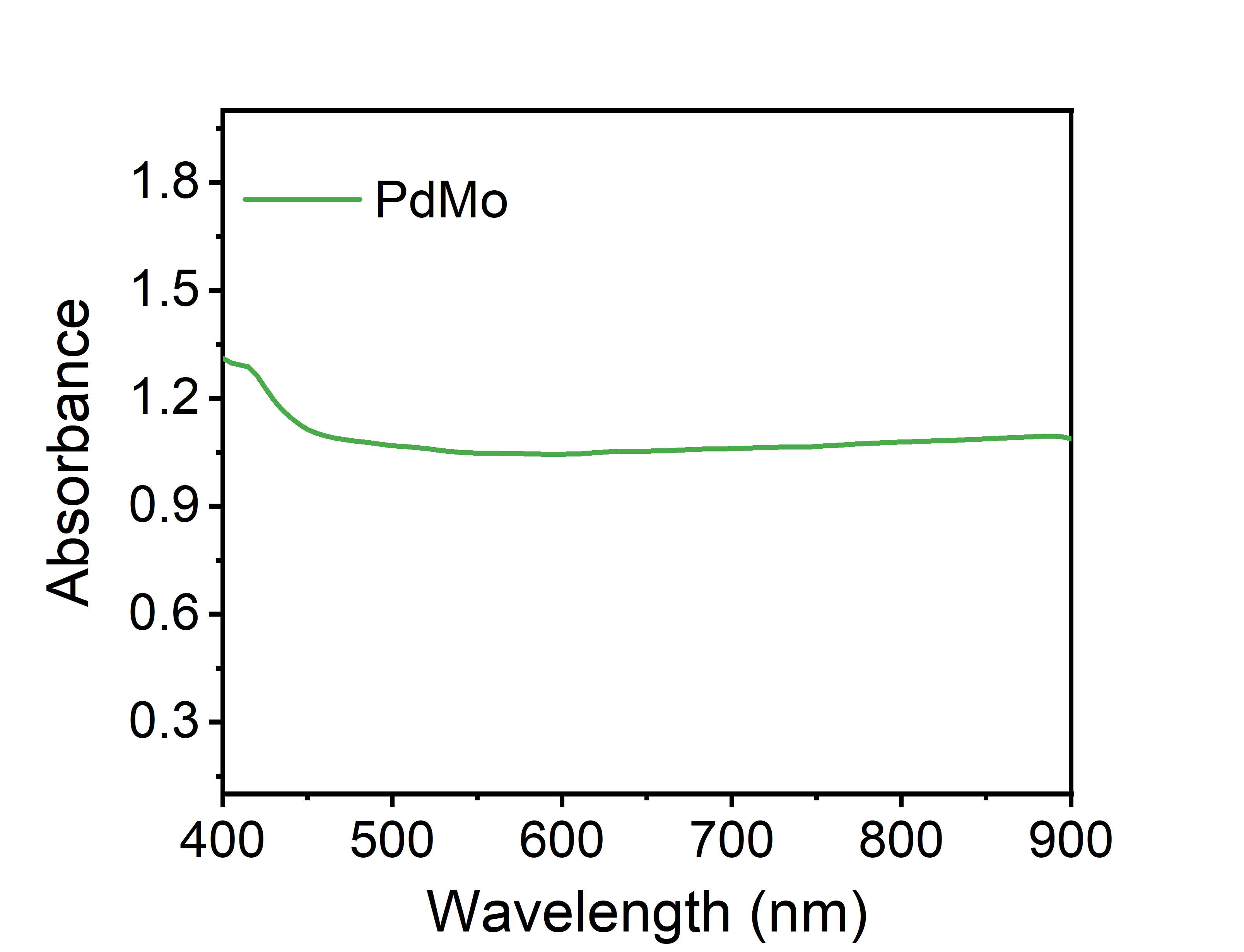


Figure S2. UV-vis absorption spectrum of PdMo nanoflowers.


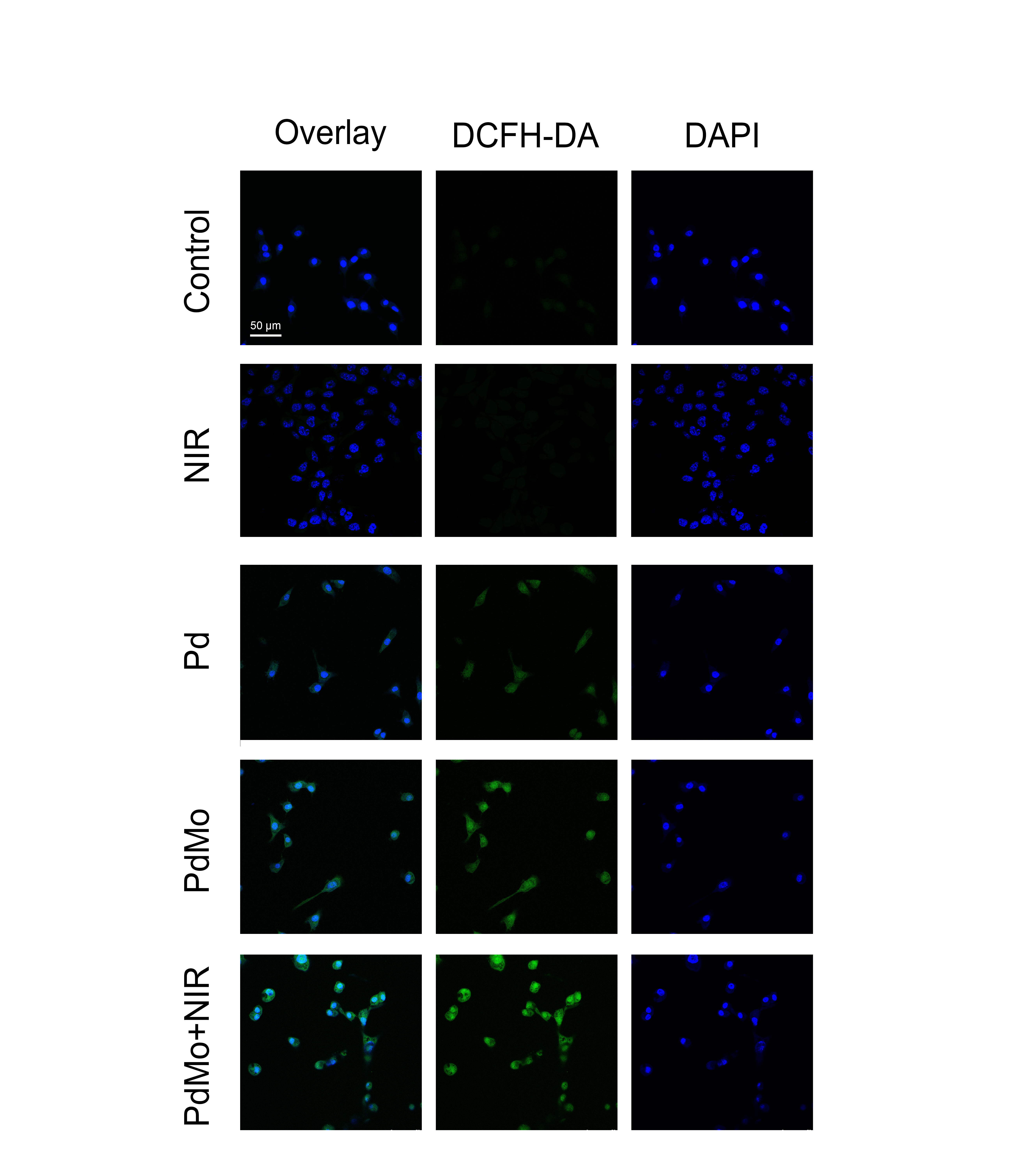


Figure S3. The intracellular ROS generation in different treatment groups measured by the ROS detection kit.


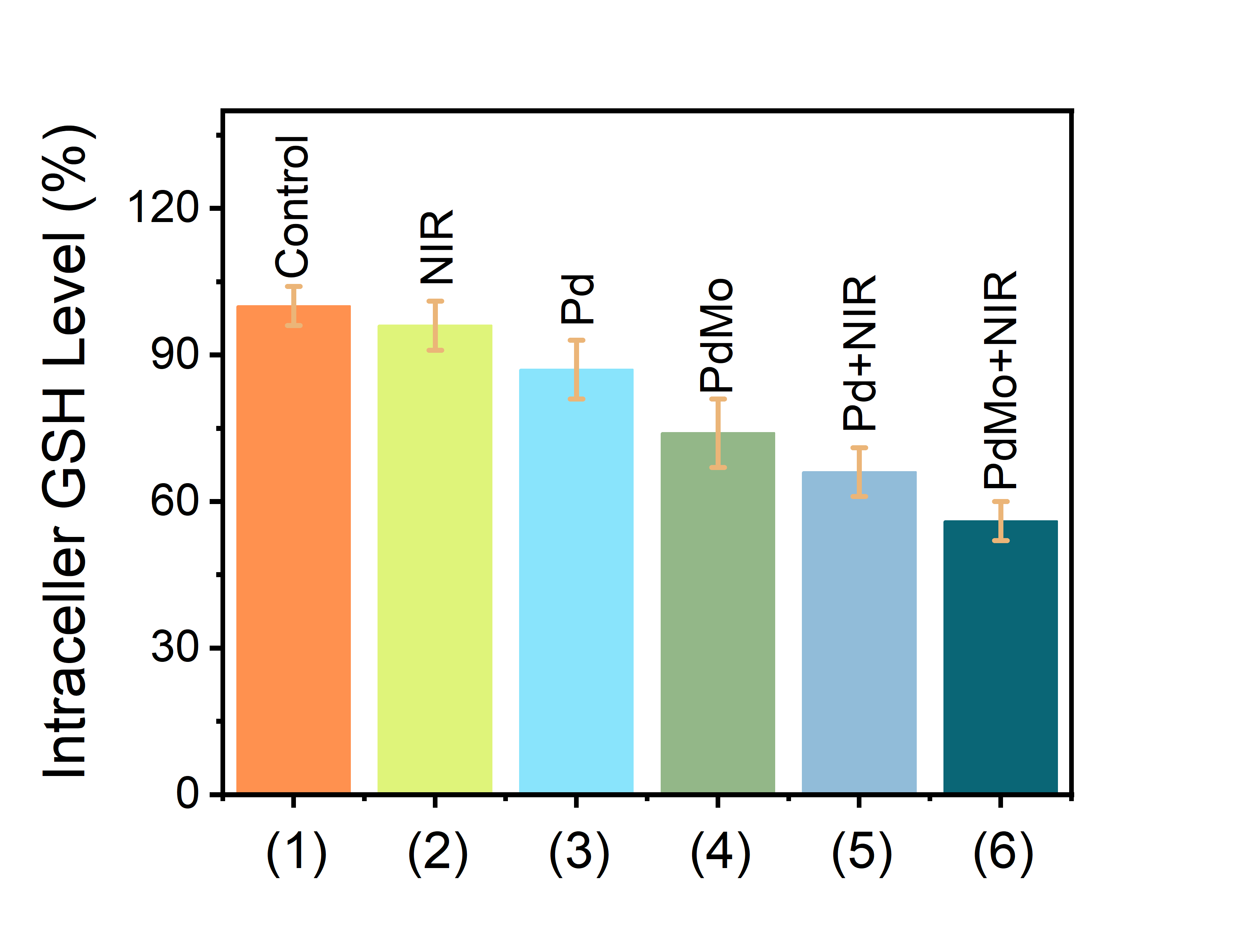


Figure S4. The intracellular GSH consumption in different treatment groups measured by the GSH detection kit


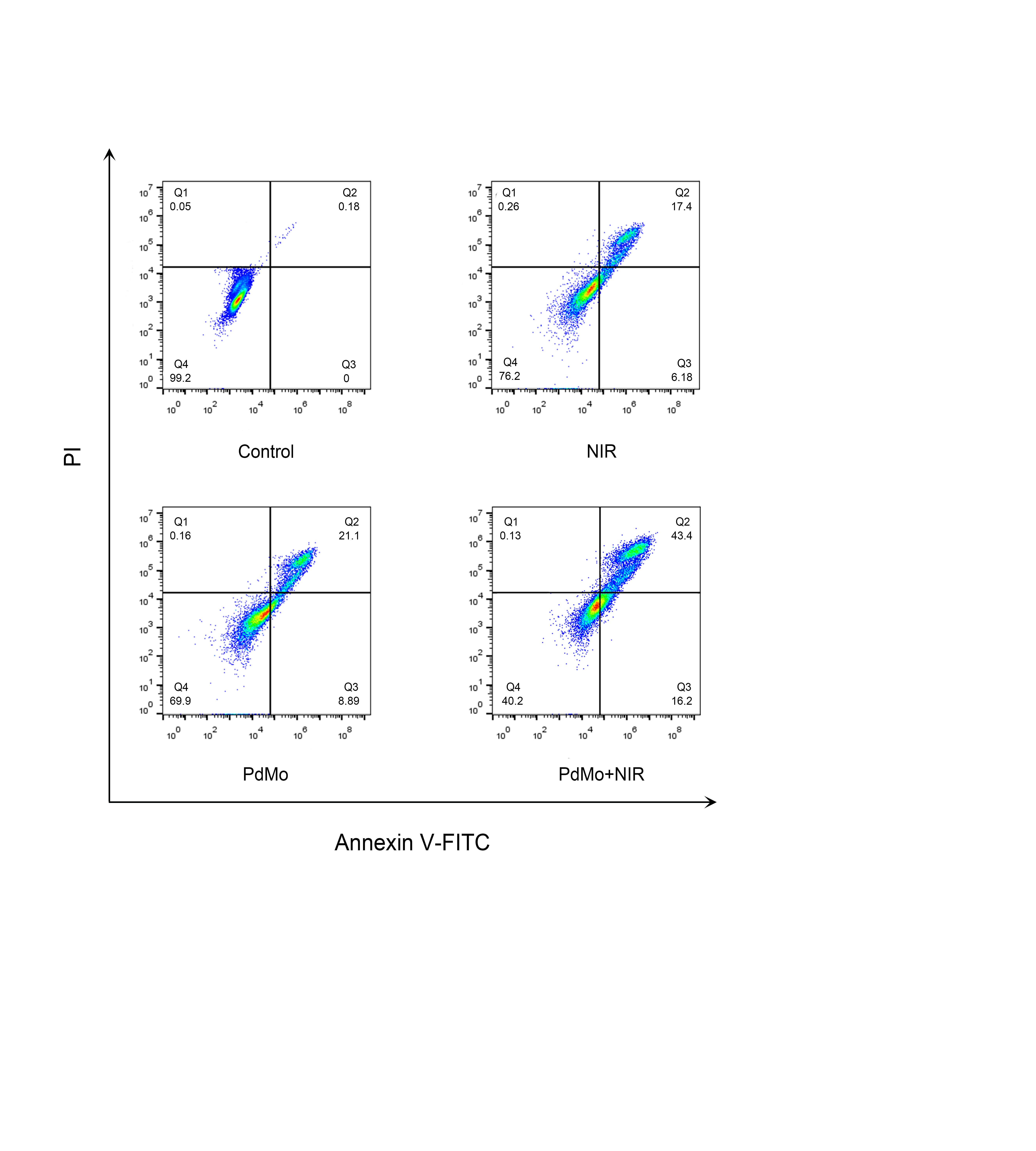


Figure S5. The apoptotic rate of CT26 cells in different treatment groups measured by flow cytometer.


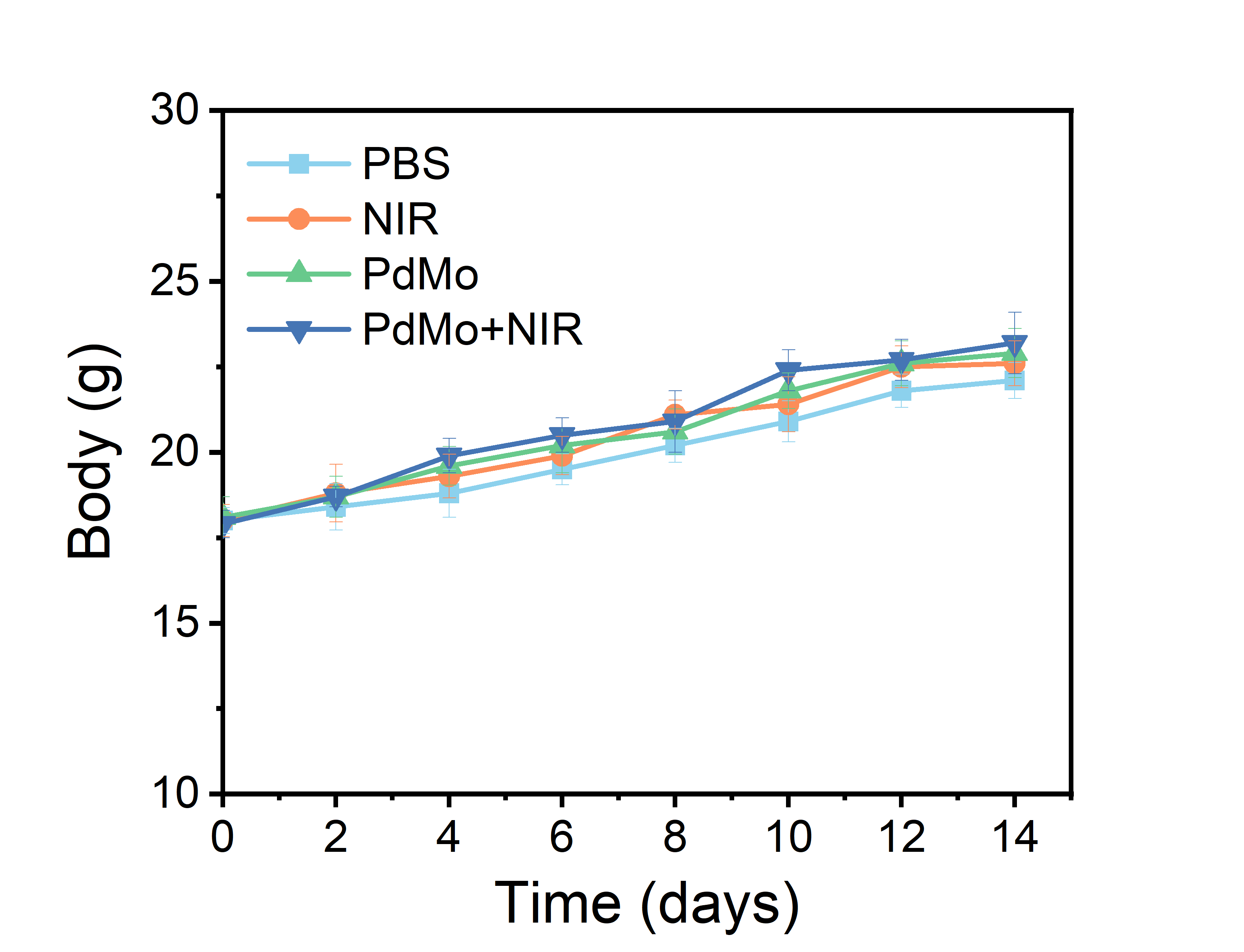


Figure S6. The body weight changes of all mice during 14-day treatments.


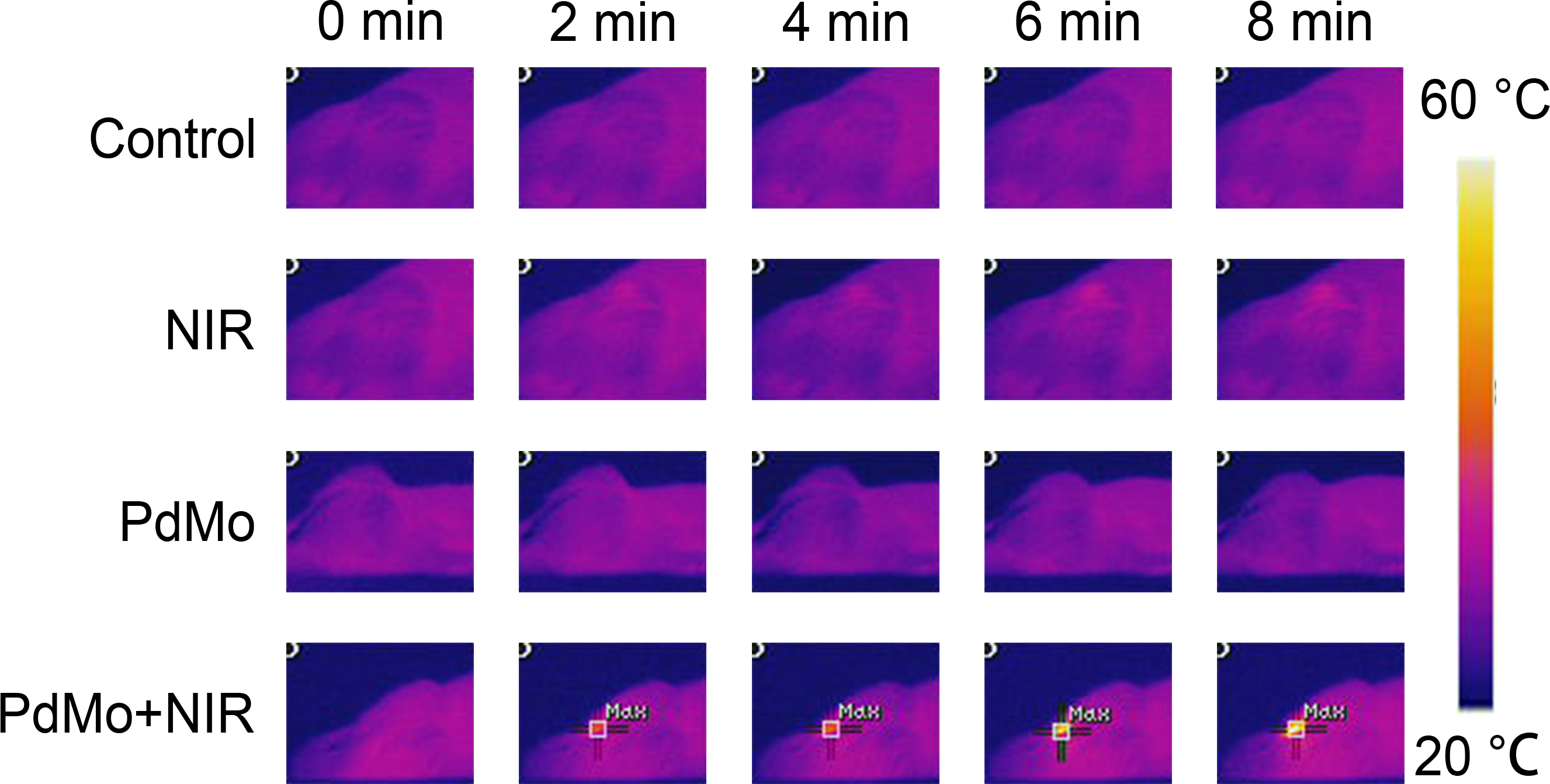

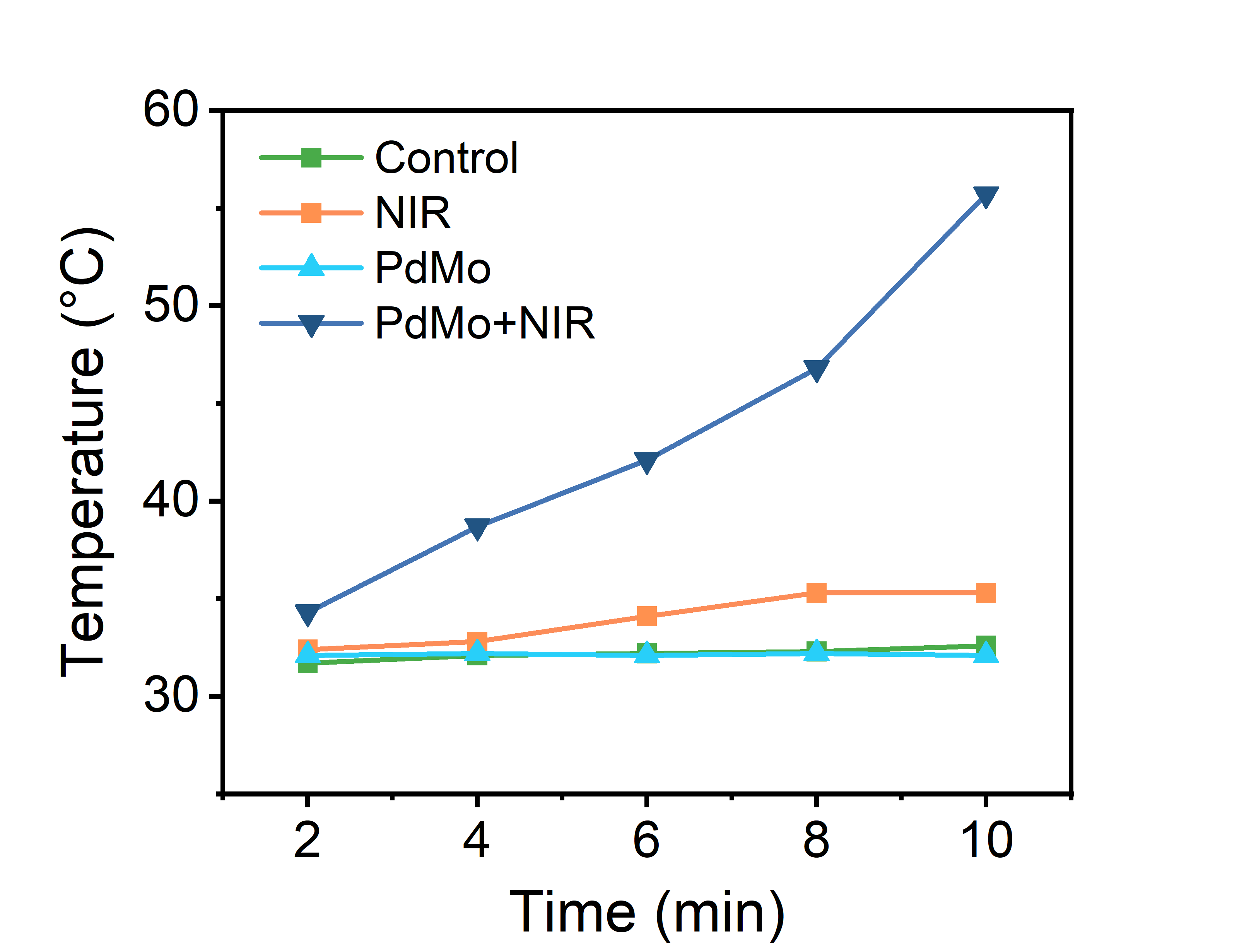


Figure S7. (a) The *in vivo* photothermal effect of PdMo nanoflowers. (b) The changes of average temperature in the tumors during NIR light irradiation in different groups.


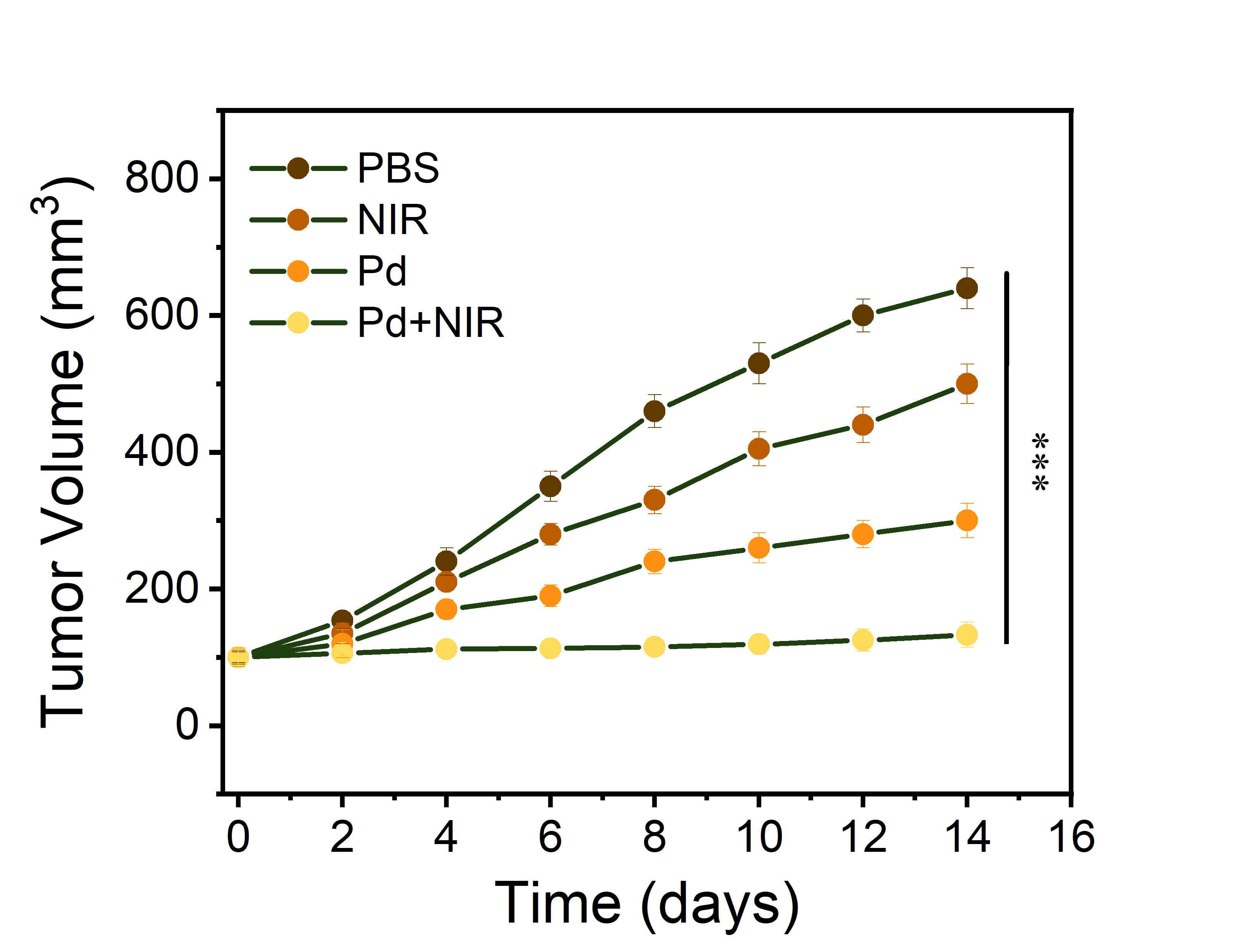

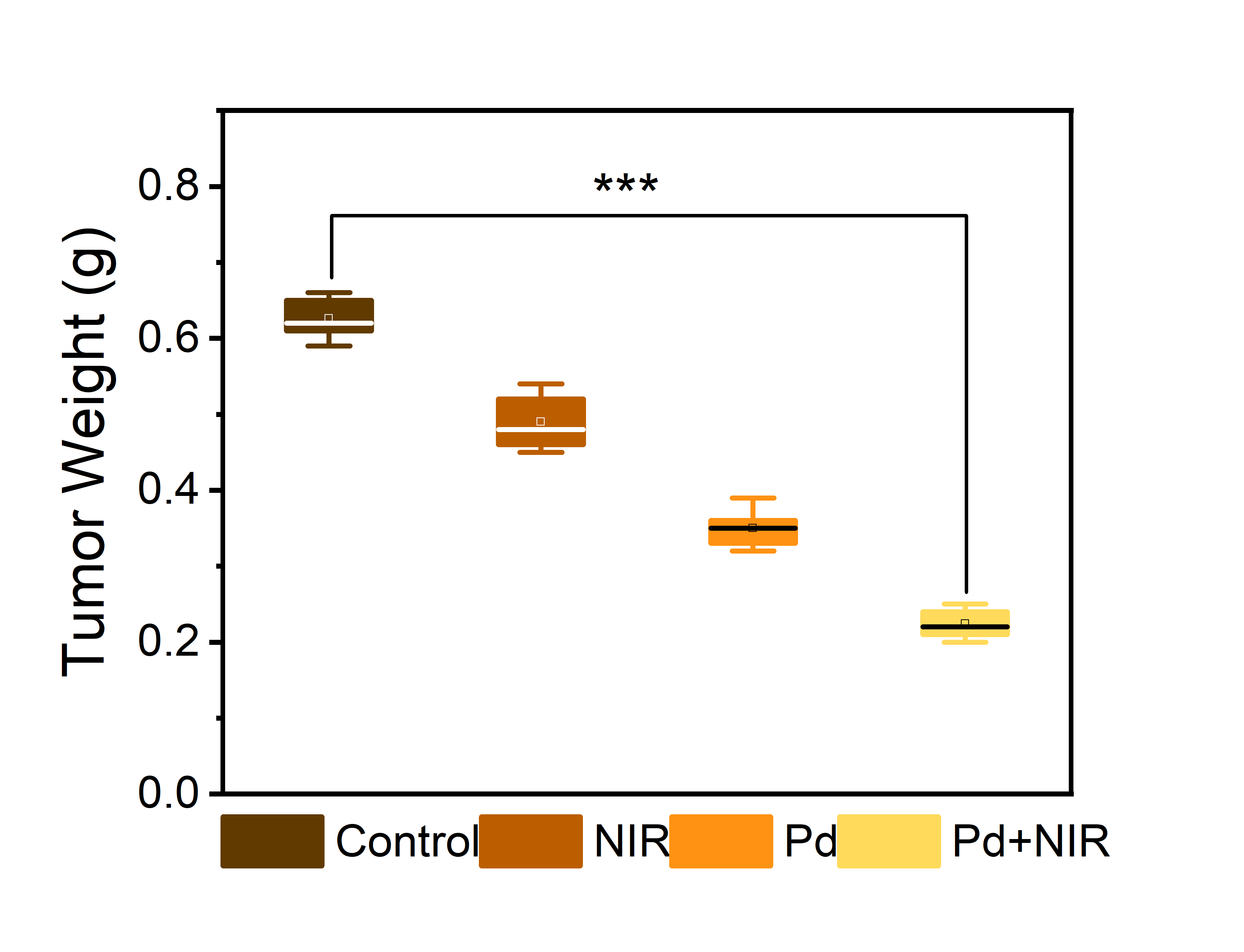


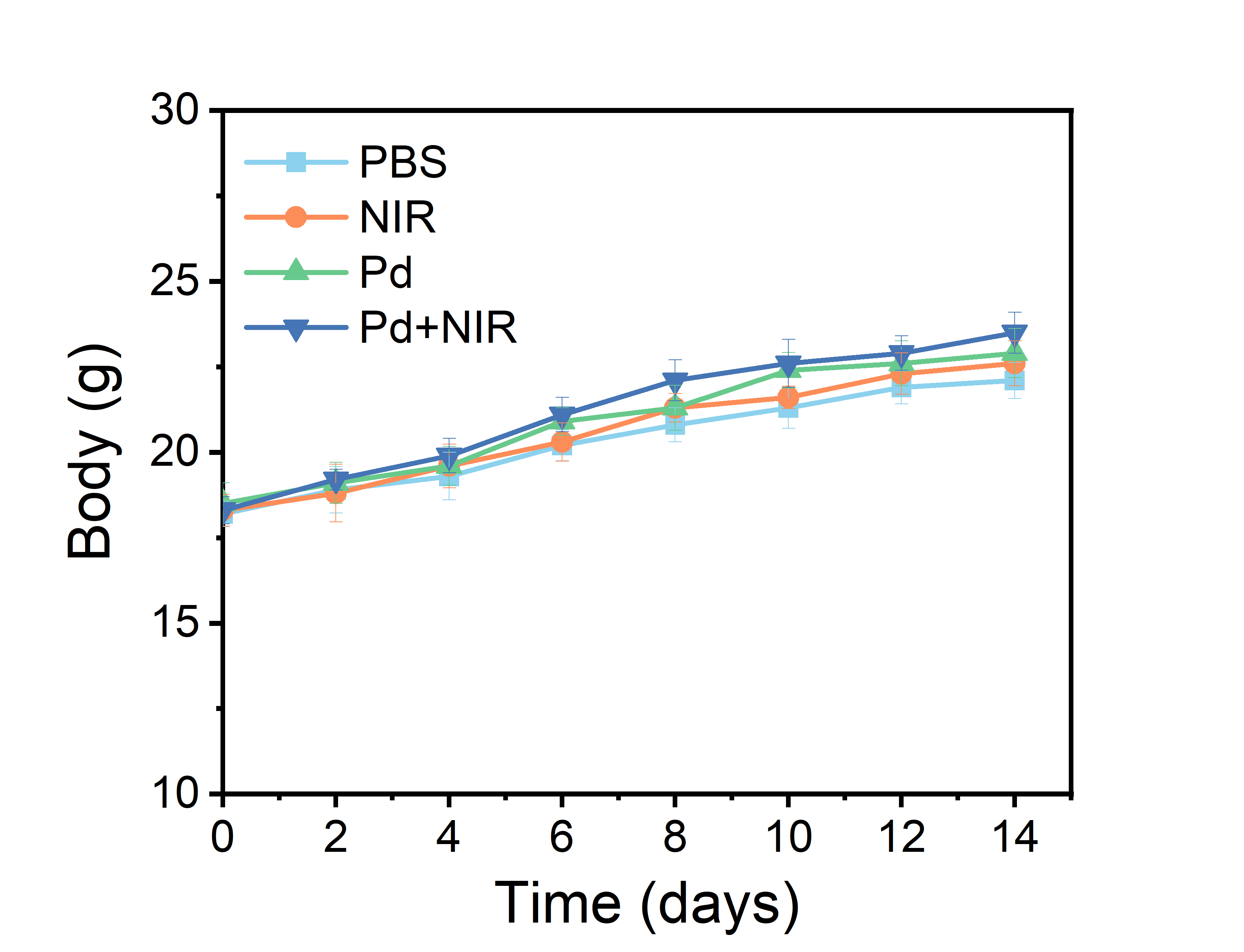


Figure S8. *In vivo* anti-tumor properties of Pd nanoalloys.


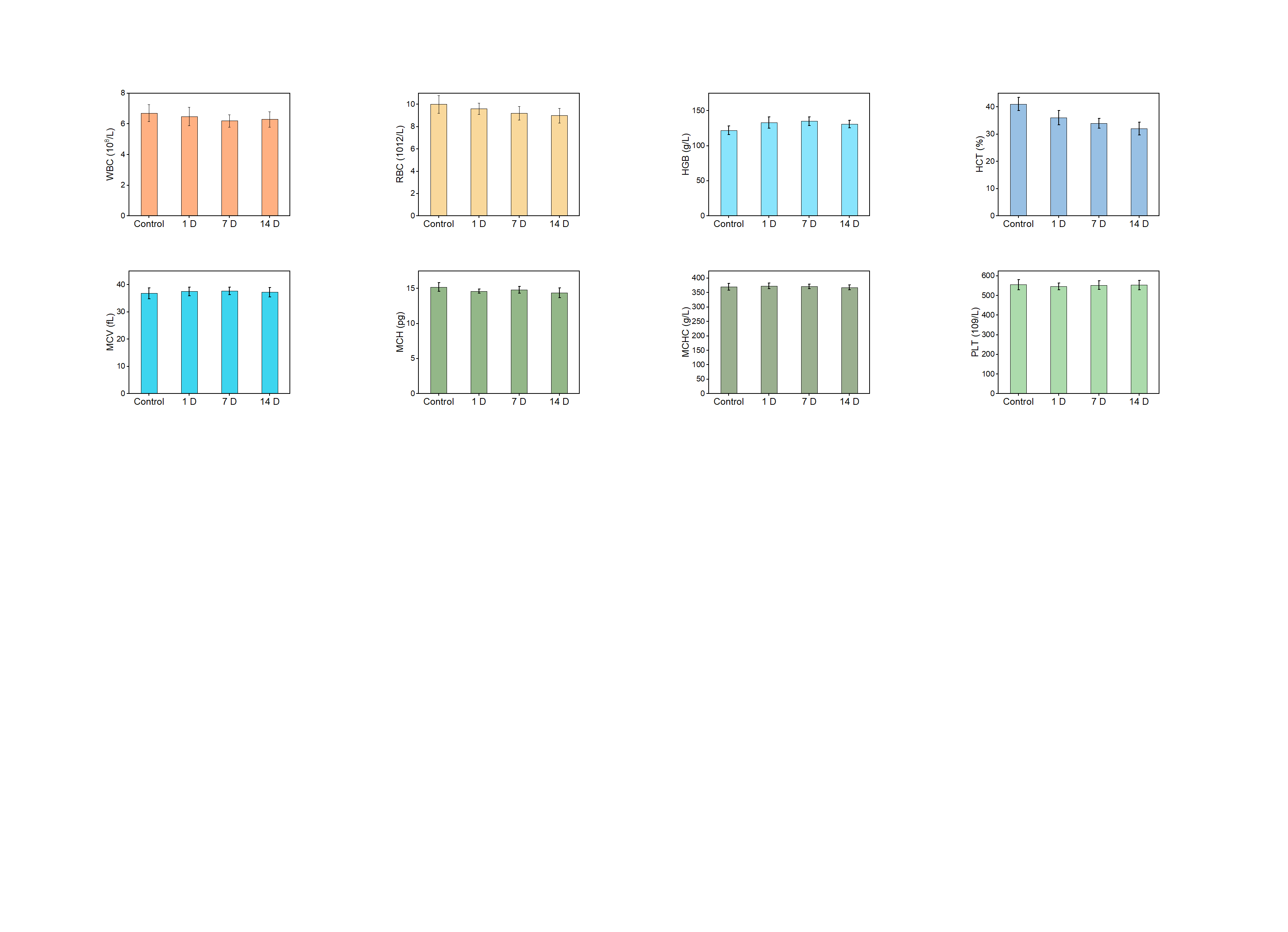


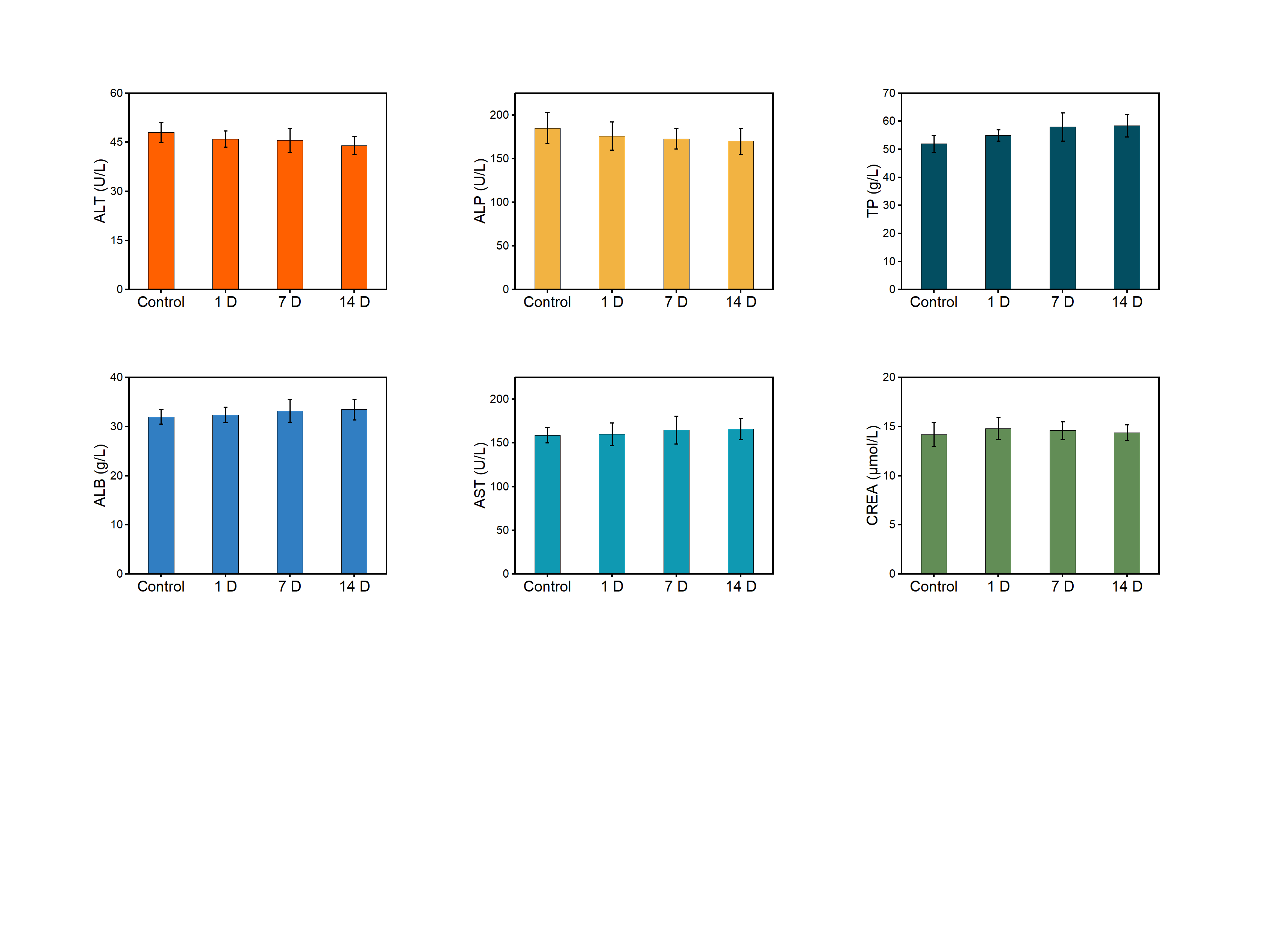


Figure S9. The blood biochemical and routine analysis of mice post 24 h and 7 day administration of PdMo nanoflowers.
